# Patient-Reported Quality of Life, Depression, Anxiety, and Physical Activity in Patients Receiving an Implantable Cardioverter-Defibrillator for Primary versus Secondary Prevention: A Single-Centre, Prospective, Observational Cohort Study

**DOI:** 10.3390/ijerph191912830

**Published:** 2022-10-07

**Authors:** Natasza Krauze, Edyta Smolis-Bąk, Ilona Kowalik, Maciej Sterliński

**Affiliations:** 1 Department of Cardiology, University Clinical Center, Medical University of Warsaw, 02-091 Warsaw, Poland; 2 Department of Coronary Artery Disease and Cardiac Rehabilitation, National Institute of Cardiology, 04-628 Warsaw, Poland; 3 Clinical Research Support Center, National Institute of Cardiology, 04-628 Warsaw, Poland; 4 1st Department of Arrhythmia, National Institute of Cardiology, 04-628 Warsaw, Poland

**Keywords:** heart failure, ICD, anxiety, depression, quality of life, physical activity

## Abstract

Study objectives: The aim of the study was to evaluate of the quality of life, depression, anxiety levels, and physical activity in the groups after the implantation of an ICD or CRT-D. Methods: All subjects (111 CHF patients) underwent tests to assess the quality of life (NHP), the level of physical activity (IPAQ), the level of perceived stress (PSS), and the incidence of depression (BDI). Results: After the implantation, physical activity (PA) of the patients from the primary prevention (PP) group remains unchanged, whereas in the secondary prevention (SP) group, it decreases noticeably. Physical activity is lower in the SP group in comparison with the PP group. There are no statistically significant differences in the level of depression. The scores in the second part of the NHP questionnaire indicate that the SP group significantly more often declare problems with housework and with social life than the PP group. Conclusions: 1. The type of prevention does not have an influence on the level of anxiety, stress, or depression. 2. The patients after implantation as SP are physically less active; lower PA is associated additionally with the higher NYHA class and chronic kidney disease. 3. The quality of life of the patients from SP group is at a lower level than patients from PP group.

## 1. Introduction

Congestive heart failure is one of the most common reasons for hospitalization of people aged over 65 years of age, and is responsible for 11% of all hospital stays. In high-income European countries, 1–2% of people (and approximately 10% of those over 70 years of age) live with heart failure [1].

The main cause of mortality in moderate congestive heart failure is sudden cardiac death. Insertion of an implantable cardioverter-defibrillator (ICD) or a cardiac resynchronization therapy defibrillator (CRT-D) can reduce the risk of sudden cardiac death and may be used in primary or secondary (i.e., in cardiac arrest survivors) prevention regimens. In addition to preventing sudden cardiac death, the insertion of a CRT-D can improve general prognosis, reduce hospitalization and mortality, increase exercise tolerance, enhance quality of life, lower the New York Heart Association (NYHA) functional class, and improve heart function in patients with intraventricular conduction abnormalities (particularly left bundle branch block, as well as symptomatic heart failure and reduced left ventricular ejection fraction) [2,3,4].

Patients with congestive heart failure often face psychological challenges. Many tools are available to evaluate psychological challenges associated with different arrhythmic problems [5]. Following the implantation of high-energy antiarrhythmic therapy devices (i.e., ICD or CRT-D), patients are particularly susceptible to emotional problems including anxiety and depression (32% of patients experience anxiety and 28% of patients have symptoms of depression) [3,6]. Physical activity (including cardiac rehabilitation) is vital for this group of people because, in addition to improving general fitness, it can have a beneficial effect on quality of life and reduce anxiety and depression [7,8,9,10].

We hypothesized that, due to their traumatic experience, patients receiving an ICD or CRT-D might adapt more easily to life with a defibrillator and, therefore, report better quality of life and lower levels of anxiety and depression compared with patients receiving an ICD or CRT-D for primary prevention. We also hypothesized that higher levels of physical activity would be associated with improved quality of life and reduced anxiety and depression among patients receiving an ICD or CRT-D.

This study aims to compare quality of life, depression and anxiety levels, and physical activity in patients undergoing implantation of an ICD or a CRT-D for primary versus secondary prevention.

## 2. Materials and Methods

This prospective observational cohort study included consecutive patients with congestive heart failure aged over 18 years with an inserted ICD or CRT-D recruited from a single center between January 2017 and December 2019. Study was approved by the local Bioethics Committee (Approval No 1372). Patients gave written, informed consent to be included in the study.

All participants underwent tests to assess psychological variables, quality of life, and level of physical activity at a minimum of 3 months following implantation. Paper forms were filled in personally by study participants, who were supported by investigators in reading, discussing, and understanding the form any time it was needed. Physical activity was scored according to the International Physical Activity Questionnaire (IPAQ; short version), which contains seven questions concerning levels of exertion within the past 7 days (vigorous-intensity, moderate-intensity, walking), as well as sitting. Participants specified whether they had performed certain activities, the number of days per week they participated in those activities, and the duration of particular physical activities within 24 h (in min) [11,12]. Participants were classified into one of the following groups on the basis of obtained scores:

- Inactive—participants are insufficiently active (have an activity level below the minimally active level);
- Minimally active—(a) 3 or more days of vigorous physical activity for 20 min per day; or (b) 5 or more days of moderate-intensity physical activity and/or a short walk for at least 30 min per day; (c) or 5 or more days of any combination, reaching at least 600 MET-min/week;
- HEPA (health-enhancing physical activity)—highly active—(a) vigorous physical activity for at least 3 days, with a minimum of at least 1500 MET-min/week; (b) 7 or more days of any combination achieving a minimum of 3000 MET-min/week [11,12].

Adopted metabolic equivalent of task (MET) values: walking 3.3 MET, moderate-intensity activity 4 MET, vigorous-intensity activity 8 MET. Calculating the level of the whole-week physical activity: MET (min/week) = walking (MET * minutes * number of days) + moderate activity (MET * minutes * number of days) + vigorous activity (MET * minutes * number of days).

Quality of life was tested with the Polish version of the Nottingham Health Profile questionnaire, adapted into native study participants’ language (Polish) by K. Wrześniewski. It consisted of two parts. The first contains 38 statements concerning energy, pain, motor limitations, and the psychosocial sphere. The second refers to the influence of current health status on the respondent’s paid work, housework (e.g., cleaning, cooking, minor repairs), social life (e.g., meeting friends, going to the cinema or theatre), family life (e.g., contact with the closest family at home), sex life, interests and hobbies (e.g., sport, DIY), and free time (e.g., holidays, weekends). Participants responded to each statement by agreeing or disagreeing that this is a particular area of concern (‘yes’ or ‘no’ answers). Scores were calculated separately for each of the six areas in the first and the second parts of the questionnaire. For part one, statistical analysis was conducted with the use of a weighted average for each question to calculate the intensity of particular features (the sum of all weighted averages in a given area equals 100) [13,14].

The level of perceived stress was assessed with the Perceived Stress Scale (PSS-10), which consists of ten questions related to subjective feelings connected with problems, personal experiences, and behaviors, as well as ways of coping with them. It is used to assess the intensity of stress connected with a patient’s life situation within the past month. The score measures chronic stress and mental wellbeing connected with the ability to cope with challenges. The range of values is 0–40 points, which are expressed in sten scores. The higher the score on the sten scale, the higher intensity of perceived stress: 1–4 stens is considered a low level of perceived stress, 5–6 stens a moderate level, and 7–10 stens a high level.

The incidence of depression and its intensity were assessed on the basis of the Polish language version of Beck’s Depression Inventory. The questionnaire consists of 21 statements concerning various symptoms of affective disorders. The participants responded to each statement by choosing the answer that—in their view—most accurately depicted their condition in the previous week. Particular questions in the Beck Depression Inventory assess both mental and somatic aspects of depression. The total score shows the severity of symptoms of depression. To analyze the obtained results, German norms were used: scores of 0–11 indicated no depression; scores of 12–19 mild depression; scores of 20–25 moderate depression; and scores of 26 or more severe depression.

Additionally, patients completed a patient report form containing 61 questions concerning personal data, medical history, type of treatment, anxiety scale (0–10 scores), disorders concerning attention span and memory, sleep disorders, and physical activity.

## 3. Results

A total of 111 patients with congestive heart failure aged 25–91 years (mean 62.6 years ± 13.4) with an inserted ICD or CRT-D were recruited between January 2017 and December 2019. Of these, 74 patients received the ICD or CRT-D for primary prevention and 37 received the ICD or CRT-D for secondary prevention. No statistically significant differences are observed between the tested groups with regard to demographic and clinical characteristics, with the exception of age, which is higher in the secondary prevention group (65.3 ± 13.2 vs. 59.6 ± 14.0; *p* = 0.043) (Table 1).

**Figure 1:**
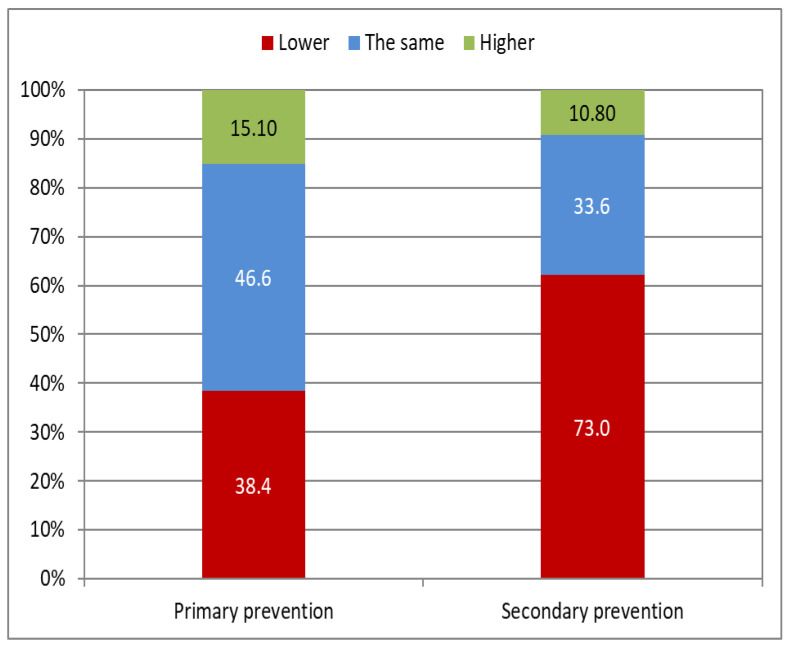
Change in physical activity after implantation, according to type of prevention.

There are no statistically significant differences in physical activity before implantation of the device (79.5% of participants in the primary prevention group vs. 83.8% in the secondary prevention group declare spending time in an active way; *p* = 0.585). After implantation, physical activity decreases in the secondary prevention group, but remains unchanged in the primary prevention group (79.4% of participants in the primary prevention group vs. 47.2% in the secondary prevention group declare spending time in an active way; *p* = 0.0006). In both groups, the preferred activities are walking and cycling; however, following implantation, the percentage of participants involved in these activities is significantly lower in the secondary prevention group (*p* = 0.016 for walking, *p* = 0.010 for cycling). Additionally, 64.9% of participants in the secondary prevention group versus 42.5% of participants in the primary prevention group (*p* = 0.026) admit that they have limited their physical activity due to fear of electrical shock, which leads to a reduction in physical activity after implantation in 73.0% of participants in the secondary prevention group versus 38.4% in the primary prevention group (*p* = 0.002) (Table 3, Figure 1).

Although the median whole-week activity in the primary prevention group (4065 MET * minutes * number of days) is 25% higher than in the secondary prevention group (3252 MET * minutes * number of days), this difference is not statistically significant (*p* = 0.717) (Table 4).

It is found that a low level of physical activity is associated with a higher NYHA classes (as the level of physical activity increases, the percentage of NYHA III or IV ambulatory patients decreases) and with the occurrence of chronic kidney diseases (Table 5).

**Figure 2:**
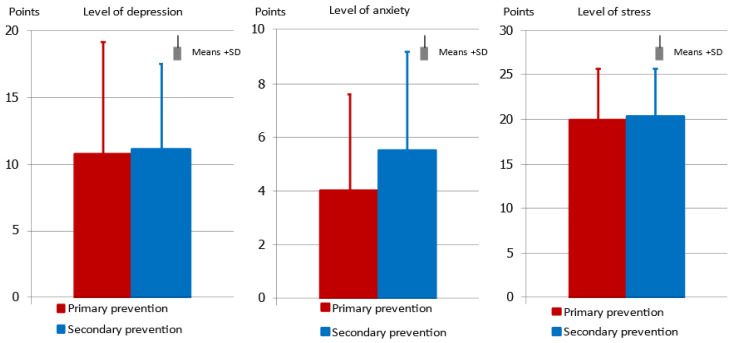
Level of depression measured by Beck’s Depression Inventory, level of anxiety measured by analogue scale, and level of stress measured by Perceived Stress Scale (PSS-10), according to type of prevention.

There are no statistically significant differences in the level of depression between the primary prevention and secondary prevention groups; there are no symptoms of depression in either group. Anxiety levels are numerically higher in the secondary prevention group than in the primary prevention group, but this difference is not statistically significant (*p* = 0.057). The groups also do not differ in terms of the severity of chronic stress connected with participants’ circumstances (Figure 2).

In the first part of the NHP questionnaire, no significant differences are observed between the study groups in all studied areas (Table 6).

**Figure 3:**
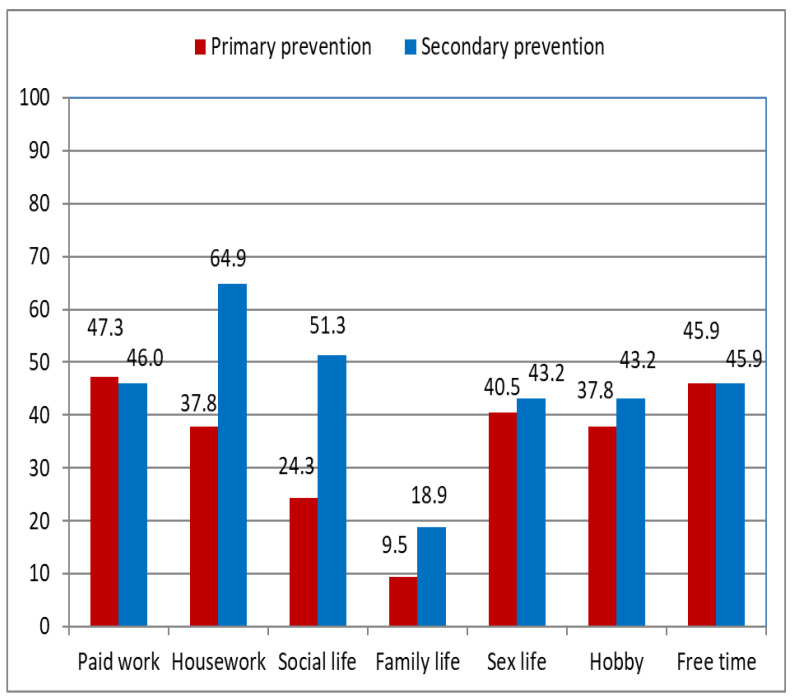
Quality of life measured using the Nottingham Health Profile questionnaire, according to type of prevention.

In the second part of the NHP questionnaire, respondents in the secondary prevention group declare problems with housework (64.9% vs. 37.8%, *p* = 0.007) and social life (51.3% vs. 24.2%, *p* = 0.004), significantly more often than respondents in the primary prevention group. Although problems connected with family life are identified in twice as many cases in the secondary prevention group, the difference is not statistically significant (9.5% vs. 18.9%) (Figure 3).

## 4. Discussion

Psychological effects in patients with heart disease are broad ranging, and evaluation must cover different areas [15]. In this prospective observational cohort study, scores assessing psychological symptoms were analyzed with reference to indications for implantation (primary vs. secondary prevention). Following the insertion of the ICD or CRT-D, we found that the physical activity levels of patients who received the device for secondary prevention are significantly lower than in patients who received the device for primary prevention.

Symptoms of depression are not observed in either group, the level of anxiety in both groups is moderate, and the level of chronic stress is between moderate and high. Anxiety levels are numerically, but not statistically significantly, higher in the secondary prevention group. In a study by Rahmawati et al. [16], in which quality of life (short form eight-item health survey), anxiety (State-Trait Anxiety Inventory), and depression (Beck’s Depression Inventory) were assessed in 179 respondents (52 participants for whom the indication for implantation was primary prevention and 127 participants for whom the indication was secondary prevention), patients in the primary prevention group experience anxiety significantly more often (*p* = 0.008) and have an inferior quality of life connected with vitality compared with patients in the secondary prevention group. No significant differences in the level of depression are identified between groups, similar to the findings of our study. Similar results are obtained in a study by Berg et al. [17], in which data obtained from 358 HeartQoL and EQ-5D questionnaires (188 patients in the primary prevention group, 167 patients in the secondary prevention group) show no difference in depression levels between groups. In a review by Freedenberg et al. [18], anxiety is present in 13–35% (24–87% less severe symptoms of anxiety) and depression in 24–33% of patients with an ICD inserted for secondary prevention. Younger individuals, women, and patients with a history of ICD shocks are particularly susceptible to psychological consequences. The work highlights the importance of screening for anxiety and depression in all candidates for an ICD, whereas cognitive behavioral therapy and psychoeducation programs might help to reduce stress in patients with an ICD and congestive heart failure [18].

Among all patients, health status appears to be related to self-reported quality of life. Nearly half of participants declare problems with paid work (47.3% in the primary prevention group vs. 46.0% in the secondary prevention group) and spending free time (45.9% in the primary prevention group vs. 45.9% in the secondary prevention group). Significantly more patients in the secondary prevention group have problems with household duties (*p* = 0.007) and social life (*p* = 0.004) than in the primary prevention group, which indicates a lower quality of life in this group. These results differ from those of an analysis of five studies by Pedersen et al. [19] in 2009, which found no difference in quality of life between those receiving implantation for primary versus secondary prevention. [18] In our study, patients in the primary prevention group indicate family life, social life, and household duties as the least limited spheres of life, whereas patients in the secondary prevention group indicate family life, sex life, and hobbies as the least affected. In a systematic review of seven randomized controlled trials (n = 5701 patients) by da Silva et al. [20], the relation between ICD shocks and quality of life is inconsistent and could depend on the interval between shocks and assessment of quality of life. There is no evidence for deterioration in quality of life in patients with an ICD, but there is a transient impairment in quality of life after electrical shocks [20].

**Study limitations**

This was an observational single-center study with a modest sample size and non-consecutive recruitment in terms of intention-to-treat. Further, there was a significant difference in the average age of the tested subpopulations. We did not collect data on electrical shocks, which could have affected quality of life.

**Clinical implications**

Our study identifies areas of inferior post-treatment adaptation in patients receiving an ICD or CRT-D for primary versus secondary prevention, highlighting areas for therapeutic intervention, including application of methods used in clinical psychology and psychotherapy. These results suggest the need for psychological well-being or pre-procedure counseling for psychological assessment.

## 5. Conclusions

1. The type of prevention: primary vs. secondary, as an indication for an implantable cardioverter-defibrillator or cardiac resynchronization therapy defibrillator does not affect levels of anxiety, stress, nor depression;
2. Patients with implantation for secondary prevention are significantly less physically active after implantation than those for primary prevention. Lower physical activity is associated additionally with the higher NYHA class and the incidence of chronic kidney disease;
3. Quality of life of patients after implantation for secondary prevention is lower than in subjects with primary prevention.

## Figures and Tables

**Table 1:** Demographic and clinical characteristics of participants, according to type of prevention.

	Primary Prevention n = 74 (66.7%)	Secondary Prevention n = 37 (33.3%)	*p* Value
Age	59.6 ± 14.0	65.3 ± 13.2	0.043
Male	63 (85.1%)	31 (83.8%)	0.852
Weight (kg)	88.7 ± 18.9	85.8 ± 14.3	0.409
Height (cm)	173.6 ± 8.3	172.8 ± 6.8	0.624
BMI (kg/m^2^)	29.3 ± 5.3	28.6 ± 3.8	0.482
Coronary artery disease	45 (61.6%)	24 (64.9%)	0.741
Myocardial infarction	43 (58.1%)	22 (59.5%)	0.892
Revascularization	28 (37.8%)	20 (54.1%)	0.104
Kidney disease	11 (14.9%)	3 (8.1%)	0.379
Dyslipidemia	12 (16.2%)	4 (10.8%)	0.445
Thyroid disease	9 (12.2%)	8 (21.6%)	0.192
DM	23 (31.1%)	3 (8.1%)	0.007
Hypertension	24 (32.4%)	13 (35,1%)	0.776
Ventricular arrhythmias	26 (35.1%)	24 (64.9%)	0.003
AF	26 (35.1%)	4 (10.8%)	0.006
Valve disorder	13 (17.8%)	3 (8.1%)	0.173
LVEF (%)	31 ± 13	34 ± 10	0.197
ICD	46 (62.2%)	33 (89.2%)	0.002
CRT-D	28 (37.8%)	4 (10.8%)	
Appropriate interventions * ICD/CRT-D	25 (33.8%)	18 (48.6%)	0.130
Inappropriate † interventions ICD/CRT-D	10 (13.5%)	4 (10.8%)	0.771
Time after implantation (years)	6.5 [5.0–9.0]	5.0 [3.0–10.0]	0.476

BMI—body mass index; LVEF—left ventricular ejection fraction; ICD—implantable cardioverter-defibrillator; CRT-D—cardiac resynchronization therapy with defibrillator. * Appropriate interventions are defined as a therapy delivered by the device as a result of ventricular tachycardia or fibrillation. † Inappropriate interventions are all device therapies delivered as a result of sinus or supraventricular tachyarrhythmia, oversensing with counter loading, or damage or interference noise. It is observed that with the NYHA class increase, the share of primary prevention increases (*p* = 0.02) (Table 2).

**Table 2:** NYHA class distribution, according to type of prevention.

	NYHA				P-Cochran Armitage Trend Test
NYHA Primary prevention	I 3 (50%)	II 40 (59.7%)	III 26 (81.2%)	IV 5 (83.3%)	0.020
Secondary prevention	3 (50%)	27 (40.3%)	6 (18.7%)	1 (16.7%)	

**Table 3:** Physical activity of patients before and after implantation, according to type of prevention.

	Primary Prevention—before Implantation n = 74 (66.7%)	Secondary Prevention—before Implantation n = 37 (33.3%)	*p* Value	Primary Prevention—after Implantation n = 74 (66.7%)	Secondary Prevention—after Implantation n = 37 (33.3%)	*p* Value
Spending time actively	58 (79.5%)	31 (83.8%)	0.585	58 (79.4%)	17 (47.2%)	0.0006
Walking	33 (44.6%)	16 (43.2%)	0.893	44 (59.5%)	13 (35.1%)	0.016
Cycling	29 (39.2%)	14 (37.8%)	0.890	19 (25.7%)	2 (5.4%)	0.010
Swimming	8 (10.8%)	3 (8.1%)	0.749	3 (4.1%)	0 (0%)	0.549
Dancing	5 (6.8%)	1 (2.7%)	0.662	2 (2.7%)	0 (0%)	0.551
Gymnastics	3 (4.1%)	2 (8.4%)	1.00	2 (2.7%)	1 (2.7%)	1.00
Running	2 (2.7%)	1 (2.7%)	1.00	0 (0%)	0 (0%)	NA
Frequency						
1–2 times per week	16 (27.6%)	8 (25.8%)	0.813	15 (25.9%)	6 (35.3%)	0.678
3–4 times per week	15 (25.9%)	10 (32.3%)		12 (20.7%)	4 (23.5%)	
Every day	27 (46.5%)	13 (41.9%)		31 (53.4%)	7 (41.2%)	
Duration						
30 min	11 (19.0%)	6 (19.3%)	0.729	14 (24.1%)	3 (17.6%)	0.708
0.5–1 h	18 (31.0%)	12 (38.7%)		18 (31.0%)	7 (41.2%)	
>1 h	29 (50.0%)	13 (41.9%)		26 (44.8%)	7 (41.2%)	

**Table 4:** Participants’ physical activity after implantation, according to type of prevention.

	Primary Prevention n = 74 (66.7%)	Secondary Prevention n = 37 (33.3%)	*p* Value
Vigorous	0 (0–480)	0 (0–0)	0.196
Moderate	390 (0–1200)	360 (0–2160)	0.545
Light (walk)	2772 (1188–4158)	2772 (693–4158)	0.528
Total	4065 (1584–8638)	3252 (2160–6426)	0.717
Activity category			
Inactive; insufficiently active	13 (17.6%)	4 (11.4%)	0.650
Minimally active; sufficiently active	19 (25.7%)	11 (31.4%)	
HEPA active; highly active	42 (56.8%)	20 (57.1%)	

HEPA—health enhancing physical activity. HEPA—health-enhancing physical activity; MET—metabolic equivalent of task.

**Table 5:** Relationship between level of physical activity, NYHA class, and comorbidities.

	Activity Category			*p*
	Inactive, Insufficiently Active	Minimally Active, Sufficiently Active	HEPA Active, Highly Active	
NYHA III or IV amb.	11 (57.9%)	9 (30.0%)	18 (29.2%)	0.042
Thyroid diseases	5 (26.3%)	5 (16.7%)	7 (11.3%)	0.112
Diabetes	6 (31.6%)	8 (26.7%)	12 (19.3%)	0.231
Chronic kidney disease	5 (26.3%)	9 (9.8%)		0.049

**Table 6:** Quality of life measured using the Nottingham Health Profile questionnaire, according to type of prevention.

	Primary Prevention n = 74 (66.7%)	Secondary Prevention n = 37 (33.3%)	*p* Value
Energy	24.0 (0–63.2)	60.8 (0.0–76.0)	0.358
Pain	0.0 (0.0–17.0)	9.0 (0.0–30.0)	0.082
Emotional reactions	9.8 (0.0–30.4)	16.5 (0.0–31.5)	0.544
Sleep disturbances	34.3 (0–77.6)	22.4 (0.0–65.1)	0.625
Social isolation	0 (0–22.0)	0 (0–22.0)	0.512
Physical mobility	21.4 (0–42.8)	21.8 (10.8–45.2)	0.352

## Data Availability

The data presented in this study are available on request from the corresponding author.
